# High-Energy Heavy Ion Irradiation of Al_2_O_3_, MgO and CaF_2_

**DOI:** 10.3390/ma15062110

**Published:** 2022-03-13

**Authors:** Juraj Hanžek, Pavo Dubček, Stjepko Fazinić, Kristina Tomić Luketić, Marko Karlušić

**Affiliations:** Ruđer Bošković Institute, Bijenička cesta 54, 10000 Zagreb, Croatia; juraj.hanzek@irb.hr (J.H.); stjepko.fazinic@irb.hr (S.F.); kristina.tomic@irb.hr (K.T.L.)

**Keywords:** ion irradiation, AFM, RBS/c, Al_2_O_3_, MgO, CaF_2_

## Abstract

High-energy heavy ion irradiation can produce permanent damage in the target material if the density of deposited energy surpasses a material-dependent threshold value. It is known that this threshold can be lowered in the vicinity of the surface or in the presence of defects. In the present study, we established threshold values for Al_2_O_3_, MgO and CaF_2_ under the above-mentioned conditions, and found those values to be much lower than expected. By means of atomic force microscopy and Rutherford backscattering spectrometry in channelling mode, we present evidence that ion beams with values of 3 MeV O and 5 MeV Si, despite the low density of deposited energy along the ion trajectory, can modify the structure of investigated materials. The obtained results should be relevant for radiation hardness studies because, during high-energy ion irradiation, unexpected damage build-up can occur under similar conditions.

## 1. Introduction

Creating defects in engineering through high-energy heavy ion beam irradiation is a well-established approach for materials modification. Ion irradiation parameters such as ion type, energy and fluence can be easily tuned to achieve the desired densities of defects at pre-defined depths [1,2,3]. Furthermore, high-energy heavy ion irradiation can also be used, with a great degree of control, to change material surfaces [4,5,6] and create nanomaterials [7,8,9,10,11]. These attractive possibilities stem from the fact that high-energy heavy ions travel through the material in almost straight lines. This way, they can penetrate several microns deep into the material, but affect only a small (around few nanometres) cylindrical region around their trajectory. These high-energy ions collide with atoms extremely rarely; hence, their main channel of energy dissipation is via numerous collisions with electrons (i.e., electronic stopping) present in the material. As a result, intense electronic excitation in the wake of the high-energy heavy ion can induce material melting locally (via electron–phonon coupling) and, as a result, an ion track (i.e., permanent damage along ion trajectory) can be formed [1,3,12,13]. 

For the ion track to be formed, the density of deposited energy should exceed a material-dependent threshold [1,3,14,15]. Otherwise, the deposited energy will simply dissipate away, leaving the material unaltered. This phenomenon often restricts research efforts and applications to radiation-sensitive materials, such as polymers and insulators, because track formation in radiation-resistant materials would require access to very high ion energies that are available only at the largest accelerator facilities. Therefore, our research is focused on finding ways to lower the threshold for ion track formation, and thus expand research possibilities available at small and medium-sized accelerator facilities. Two approaches relevant for the present study are ion irradiation at grazing incidence angles and sequential ion irradiations.

In several studies, we have shown that ion tracks can be produced on material surfaces much more easily than in their bulk. For example, we have established a threshold for ion track formation in the bulk of SrTiO_3_ at 12 keV/nm, and on the surface it was around 7 keV/nm [16,17]. Likewise, in the case of GaN, tracks have not been produced in the bulk after 90 MeV Xe irradiation (23 keV/nm), but small tracks have been found on the surface after 23 MeV I irradiation (8 keV/nm) [18]. Most recently, we have also found that ion tracks can be found on surfaces of highly resistant oxides Al_2_O_3_ and MgO already at 8–9 keV/nm, well below the threshold for ion track formation in the bulk, which is considered to be between 15 and 18 keV/nm [19]. Therefore, the first aim of the present study is to find the threshold for surface ion track formation in Al_2_O_3_, MgO and CaF_2_, using ion beams with low values of electronic stopping and negligible nuclear stopping contribution. The later contribution could be important to establish a value close to the threshold, since it can influence damage production [20], but this is beyond the scope of this work.

Sequential ion irradiations have also attracted a lot of interest recently because coupling between nuclear and electronic stopping can be studied with this approach. For example, in the case of SrTiO_3_, the introduction of defects using low-energy irradiation (i.e., via nuclear stopping) made this material more susceptible to ion track formation. It was shown that the irradiation of the damaged SrTiO_3_ with the high-energy heavy ion beam with an electronic stopping point below the nominal threshold (10 keV/nm) still resulted in the formation of ion tracks with 2 nm in diameter [21]. Sequential irradiation studies on SiC have shown the opposite behaviour: that defects produced by low-energy irradiation can be effectively erased by high-energy irradiation (i.e., via electronic stopping) [22,23]. An extremely low value of 2 keV/nm for electronic stopping has been found to be sufficient for the recovery of SiC crystal, otherwise known as extremely radiation-resistant material with a threshold for ion track formation above 34 keV/nm [24]. Most recently, in our study of sequential ion irradiation of silicon, the removal of defects has been found after irradiation with a 23 MeV I beam (5 keV/nm). This is in contrast to previous works, whereby track formation in undamaged silicon has been found only after cluster ion irradiations [25,26,27]. Thus, the second aim of this work was to investigate Al_2_O_3_, MgO and CaF_2_ materials’ behaviour when subjected to sequential heavy ion irradiation.

## 2. Experimental Details

Single-crystal samples of MgO (100) and Al_2_O_3_ (0001) have been purchased from MaTecK ( Jülich, Germany) and CrysTec ( Berlin, Germany), respectively. The Al_2_O_3_ samples were 5 mm × 5 mm in size, and the MgO samples were 7 mm × 7 mm in size. Their thickness was 0.5 mm and the surfaces were epi-polished. The CaF_2_ (111) samples were cleaved prior to the irradiation from the single-crystal piece purchased from Korth (Altenholz, Germany). The size of CaF_2_ samples was 7 mm × 7 mm, with a thickness up to 1 mm. No further sample preparation has been performed before the irradiations.

All samples were irradiated using 6 MV EN Van de Graaff accelerator (HVEC, Burlington, MA, USA) at the Ruđer Bošković Institute in Zagreb. In Table 1, all ion beams used in this work have been listed. For the precise sample positioning, a 4-axis goniometer mounted at the ToF-ERDA beamline was used [28]. The ion beam was scanned using 2 pairs of magnetic coils positioned 4 m in front of the chamber. Applied ion fluences were evaluated by measuring the ion current before and after each exposure. For the highest fluences, additional current measurements were performed when necessary. Samples have been irradiated both at the normal and grazing incidence orientations. Samples irradiated at the normal incidence were tilted by 5° off normal to avoid possible heavy ion channelling, and samples irradiated at the grazing incidence were positioned at a 1.5° incidence angle with respect to the surface.

The effects of ion irradiation on samples irradiated at the normal incidence were investigated using Rutherford backscattering spectrometry in channelling mode (RBS/c). For this analysis, we have used a 1 MeV proton beam delivered from a 1 MV Tandetron accelerator. Samples were mounted on the 5-axis goniometer, and their alignment was performed by obtaining angular scan maps (azimuth, tilt). The beam spot size was 1 mm, and the current was kept at a few nA to avoid possible interferences with data acquisition. For the detection of backscattered protons, a silicon surface barrier detector was positioned at 160° with respect to the incoming beam.

The surfaces of samples irradiated at a grazing incidence angle have been inspected using atomic force microscopy (AFM). This was performed in contact mode, using NTEGRA Prima AFM (produced by NT-MDT spectrum instruments). All the collected images are 1 µm by 1 µm, 512 by 512 pixels. In the analysis, we applied the Gwyddion code [29], where only basic scan levelling and scratch filtering was performed. Before the examination, CaF_2_ samples were moderately heated (up to 200 °C) to remove water adsorbates accumulated during storage in ambient conditions.

## 3. Results and Discussion

### 3.1. Ion Irradiation Effects in Al_2_O_3_

**Figure 1:**
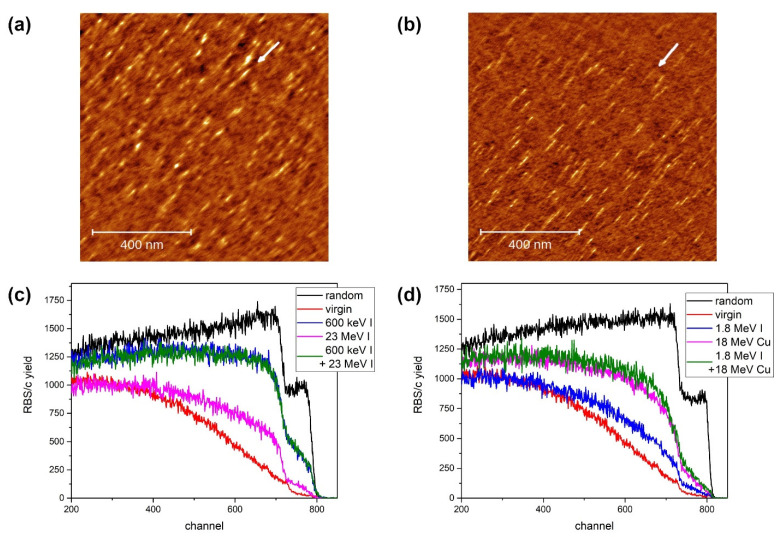
AFM images (1 µm × 1 µm, 1 nm false colour height scale) of ion tracks on Al_2_O_3_ surface irradiated with (**a**) 5 MeV Si and (**b**) 12 MeV Si ion beams at a grazing incidence angle of 1.5°. The ion beam direction is indicated by an arrow. (**c**) RBS/c spectra of sequentially irradiated Al_2_O_3_ with 600 keV I (10^14^ ions/cm^2^) and 23 MeV I (10^14^ ions/cm^2^). (**d**) RBS/c spectra of sequentially irradiated Al_2_O_3_ with 1.8 MeV I (3 × 10^13^ ions/cm^2^) and 18 MeV Cu (5 × 10^13^ ions/cm^2^).

In our previous study, we found ion tracks on the Al_2_O_3_ surface after a grazing incidence irradiation of 23 MeV I [19]. In Figure 1a,b, we present evidence that surface ion tracks can be found also after 12 MeV Si and even lower at 5 MeV Si beam irradiation. Electronic stopping powers for these two ion beams are 5.9 keV/nm and 4.6 keV/nm, respectively. These values are much lower than the 9 keV/nm electronic stopping power value of 23 MeV I from our previous study, from which an image of an unirradiated surface has also been shown [19]. Here, we also observe well-developed, although rather short ion tracks. This result sets the upper limit of the threshold for surface ion track formation at 4.6 keV/nm, which is much lower than the previously reported value of 10 keV/nm (both for surface and for bulk ion track formation) after normal incidence irradiation [30,31,32,33]. Prompt recrystallisation has been proposed as a mechanism that makes Al_2_O_3_ resistant to ion track formation, and molecular dynamics (MD) simulations indeed demonstrated this effect [19,34]. Since the vicinity of the surface breaks the cylindrical symmetry around the ion track produced by grazing incidence irradiation, recrystallisation is suppressed and tracks are formed much more easily. Hence, the results presented here clearly demonstrate the suppression of recrystallisation and could easily be used for testing of the before-mentioned MD simulations.

The surface damage caused by 5 MeV and 12 MeV Si ion beams at a grazing incidence angle of 1.5° are shown in Figure 1a,b, respectively. Rather short (30–50 nm) tracks have been formed after both energy irradiations. The tracks’ maximum height is less than 1 nm. Judging from the track density, the efficiency of their formation does not change with the ion beam energy. Regarding the apparent difference in track widths, it is not clear yet if it depends on the energy, or if it was an artefact of the scanning: since the experiment was performed in contact mode, different smearing due to the AFM tip passage is possible. Nevertheless, ion tracks are readily formed by both 5 MeV Si and 12 MeV Si irradiations (4.6 keV/nm and 5.9 keV/nm, respectively), significantly below the track formation threshold which was established previously at 9 keV/nm [19].

The effects of sequential ion irradiation were investigated using RBS/c, and the results are shown in Figure 1c,d. Low-energy ion irradiation (either 600 keV I or 1.8 MeV I) was used to produce damage in Al_2_O_3_ via nuclear stopping. Rather high applied ion fluences (10^14^ ions/cm^2^ and 3 × 10^13^ ions/cm^2^ for 600 keV I and 1.8 MeV I, respectively) resulted in a moderate amount of damage. Only for the 600 keV I exposure, i.e., the highest applied fluence and the highest nuclear stopping, is the backscattering of protons from aluminium observed, indicating significant disordering of the material. Irradiation with high-energy ions (23 MeV I at a fluence of 10^14^ ions/cm^2^ and 18 MeV Cu at a fluence of 5 × 10^13^ ions/cm^2^) resulted in the production of point-like defects only, mostly in oxygen sublattice. Both high-energy ion beams have electronic stopping of 9 keV/nm, which is supposed to be below the threshold for ion track formation, which is at 10 keV/nm [30,31,33]. Therefore, these ion beams should be excellent for the observation of defect annealing via electronic stopping. However, the results presented here suggest that this is not the case. Neither the annealing of defects nor the increased sensitivity of damaged material to ion track formation was observed.

### 3.2. Ion Irradiation Effects in MgO

**Figure 2:**
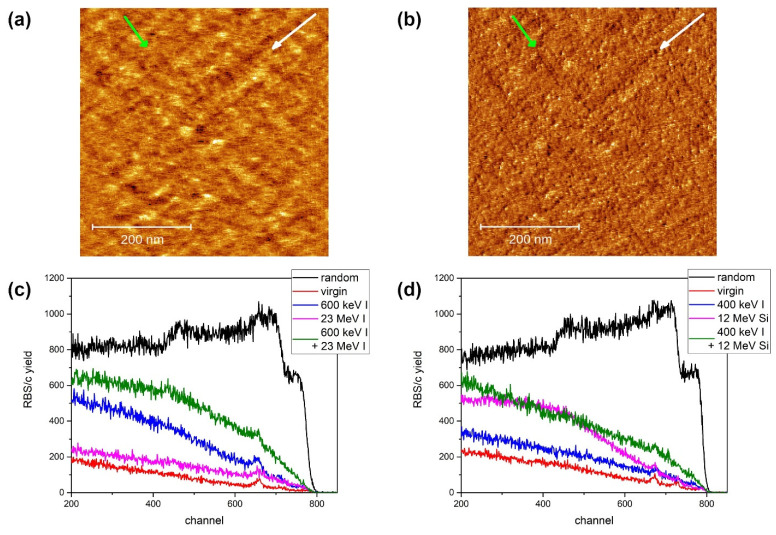
AFM image (500 nm × 500 nm, 1 nm false colour height scale) of MgO surface irradiated with 5 MeV Si (direction indicated by green arrow) and 12 MeV Si (direction indicated by white arrow) ion beams at a grazing incidence angle of 1.5° (**a**) and the corresponding friction map (**b**,**c**) RBS/c spectra of sequentially irradiated MgO with 600 keV I (10^14^ ions/cm^2^) and 23 MeV I (10^14^ ions/cm^2^). (**d**) RBS/c spectra of sequentially irradiated MgO with 400 keV I (10^14^ ions/cm^2^) and 12 MeV Si (3 × 10^15^ ions/cm^2^).

Similar to the Al_2_O_3_, well-developed ion tracks (albeit with a groove-like morphology) have also been found recently on the surface of MgO after irradiation with 23 MeV I at a grazing incidence angle [19]. In that case, the electronic stopping of 8 keV/nm was much lower than the previously reported thresholds for ion track formation of between 15 and 18 keV/nm [31,32,34,35,36]. Here, as shown in Figure 2a,b, we present evidence of surface ion track formation for even lower value of electronic stopping power. The irradiation was performed on a single sample, but at right angles to each other. Tracks were found at 5.6 keV/nm, i.e., after 12 MeV Si irradiation. The observed ion track morphology is again groove-like, but due to the threshold proximity, height variations of less than 1 nm were found. Presumably, the much lower electronic stopping value of 12 MeV Si does not excite the material as much as the 23 MeV I beam and, as a result, only faint ion tracks can be found, which are close to the limit of detection. In the case of 5 MeV Si irradiation, i.e., at 4.2 keV/nm, topological evidence of ion tracks is practically invisible. However, the material structure at the surface is slightly changed by the ion passage, which is revealed as a change of interaction with the AFM tip. This local change in interaction can be seen as streaks in the friction map. The white and green arrows in Figure 2a,b point at the chosen ion tracks formed by 12 MeV and 5 MeV Si irradiation, respectively. While the track formed by 12 MeV Si irradiation can readily be seen on both topological (Figure 2a) and friction (Figure 2b) maps, the 5 MeV Si track is virtually invisible in the topological map. Therefore, we consider these findings to be evidence that the threshold for surface ion track formation in MgO exists between 4.2 and 5.6 keV/nm. This is also in agreement with images of irradiated and unirradiated MgO surfaces presented in our previous work [19].

The RBS/c investigation of sequential ion irradiation effects reveals that MgO can be made to be more susceptible to high-energy ion irradiation by introducing defects via nuclear stopping. After high-fluence (10^14^ ions/cm^2^) irradiation with keV iodine ions, we observed low levels of defects introduced in the material. However, even this small number of defects play an important role in sensitizing MgO to the effects of high-energy ion irradiations. Both 23 MeV I and 12 MeV Si, with electronic stopping values of 8.2 keV/nm and 5.6 keV/nm, respectively, are significantly below the threshold for ion track formation (reportedly between 15 and 18 keV/nm). Therefore, only a small number of point-like defects are produced after irradiations with high fluences of 10^14^ ions/cm^2^ and 3 × 10^15^ ions/cm^2^, respectively, resulting only in a slight de-channelling of the analysing proton beam. However, in both cases of sequential ion irradiation, pronounced damage build-up is visible as a signature of synergistic effects. Previously, this type of behaviour had been observed only for a much higher value of electronic stopping power (11 keV/nm) when a 36 MeV W beam has been used [37]. Our results indicate that this effect occurs also at much lower values of electronic stopping power (5.6–8.2 keV/nm), although the applied fluences have to be large. Furthermore, ion beam flux can also play a role, as simultaneous ion irradiation is known to produce the opposite effect [38].

### 3.3. Ion Irradiation Effects in CaF_2_

**Figure 3:**
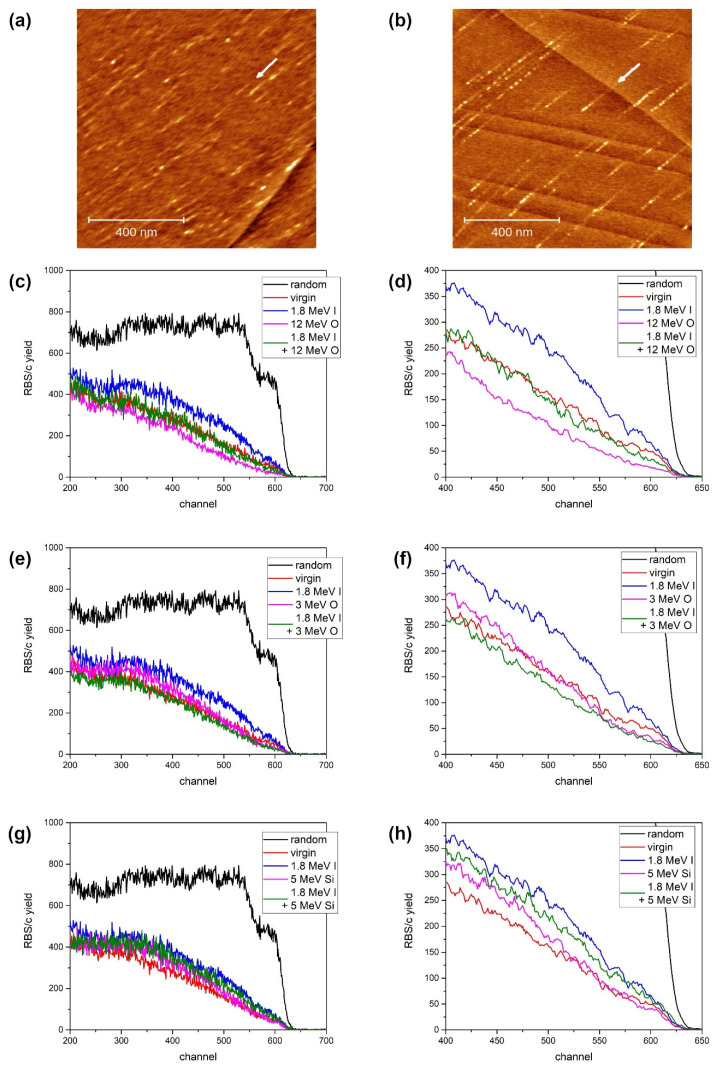
AFM images (1 µm × 1 µm, 1 nm false colour height scale) of ion tracks on CaF_2_ surface irradiated with (**a**) 5 MeV Si and (**b**) 12 MeV Si ion beams at a grazing incidence angle of 1.5°. The ion beam direction is indicated by an arrow. (**c**) RBS/c spectra of sequentially irradiated CaF_2_ with 1.8 MeV I (6 × 10^13^ ions/cm^2^) and 12 MeV O (10^14^ ions/cm^2^). (**d**) Same spectra shown between 400 and 650 channels, and smoothened with a moving average of 5 channels. (**e**) RBS/c spectra of sequentially irradiated CaF_2_ with 1.8 MeV I (6 × 10^13^ ions/cm^2^) and 3 MeV O (10^14^ ions/cm^2^). (**f**) Same spectra shown between 400 and 650 channels, and smoothened with a moving average of 5 channels. (**g**) RBS/c spectra of sequentially irradiated CaF_2_ with 1.8 MeV I (6 × 10^13^ ions/cm^2^) and 5 MeV Si (10^14^ ions/cm^2^). (**h**) Same spectra shown between 400 and 650 channels, and smoothened with a moving average of 5 channels.

In another previous study, we showed that ion tracks on CaF_2_ surfaces can be found after exposure to 10 MeV I beam when irradiated at the grazing angle of incidence [39]. Despite the low value of 2.6 keV/nm electronic stopping power, this particular ion beam also has an additional 0.6 keV/nm nuclear stopping contribution. Therefore, to establish reliably the threshold for ion track formation on the CaF_2_ surface, formed after grazing incidence irradiation, we have repeated the experiment using 12 MeV Si and 6 MeV Si beams. These ion beams have electronic stopping values of 4.5 keV/nm and 3.3 keV/nm, respectively, while their nuclear stopping values are negligible (0.02 keV/nm and 0.03 keV/nm, respectively). As shown in Figure 3a,b, ion tracks can be found by means of AFM after either irradiation. These tracks are small and less than 1 nm in height. In addition, the 5 MeV Si tracks are considerably shorter, but the overall efficiency of track formation appears to be similar for both energies. Since ion tracks and defects are known as sites for adsorbates agglomeration [40,41,42], we found that heating the samples up to 200 °C was sufficient for their effective removal before the AFM measurements. The obtained AFM images present further evidence that the threshold for surface ion track formation after grazing incidence ion irradiation is much smaller than the threshold for ion track formation after normal incidence irradiation, which was established at 5 keV/nm for CaF_2_ [43,44]. In another study [45], the threshold for ion track appearance has been estimated to be around 2.8 keV/nm because 12 MeV Xe ion impacts produced surface tracks. However, this value might have been an underestimate because the nuclear stopping contribution of 0.6 keV/nm has not been taken into account [46].

To investigate the effects of the sequential ion irradiation of CaF_2_, the first goal was to establish high-energy ion irradiation conditions when ion tracks are not formed. In our previous study [39], we observed damage build-up after 10 MeV I irradiation. Despite the low value of electronic stopping in that case, there was also a significant contribution from nuclear stopping (2.9 keV/nm and 0.6 keV/nm, respectively). Considering the high efficiency of damage formation that nuclear stopping has [9,46,47], for the present study, it was necessary to use MeV ion beams with negligible nuclear stopping. Therefore, we have used 3 MeV O, 12 MeV O and 5 MeV Si beams, thus covering the range of electronic stoppings between 2.1 and 3.3 keV/nm. Each of these ion beams has nuclear stopping values two orders of magnitude smaller than the respective electronic stopping. Furthermore, a 12 MeV O beam could also benefit from the velocity effect [7], although it is an open question whether this effect is active in CaF_2_ or not [48,49].

As shown in Figure 3c–g, the electronic stopping of all these ion beams (3 MeV O, 12 MeV O and 5 MeV Si) is below the threshold for ion track formation. Actually, irradiation with the 12 MeV O beam improves crystal quality, since RBS/c spectra show better channelling than in the unirradiated sample. We relate this observation to the fact that the presence of intrinsic defects can slightly influence channelling. This is the reason why the annealing of the samples at 400 °C before the experiment is sometimes performed in order to improve their crystallinity [44]. Sequential ion irradiation was accomplished by irradiating samples first with a 1.8 MeV I beam to the fluence of 6 × 10^13^ ions/cm^2^. This first step introduced a moderate number of defects, as shown in the related RBS/c spectra. Then, in the next step, CaF_2_ crystals were exposed to the before-mentioned high-energy ion beams (3 MeV O, 12 MeV O and 5 MeV Si), to the fluence of 10^14^ ions/cm^2^. The RBS/c analysis provided evidence that damaged crystals recovered completely after 3 MeV O and 12 MeV O irradiations. The case of 5 MeV Si irradiation appears to be a borderline case, because only partial recovery was found. Considering its highest electronic stopping, probably already close to the ion track formation threshold, it seems that the production of point-like defects is balanced with the annealing of the existing defects. However, it is clear that, even in this case, there is no additive coupling between 1.8 MeV I and 5 MeV Si irradiations.

## 4. Conclusion

In this experimental study, we have investigated the response of Al_2_O_3_, MgO and CaF_2_ to high-energy heavy ion irradiation. By means of AFM, ion tracks have been found on the surfaces of Al_2_O_3_ and CaF_2_ after 5 MeV Si irradiation, and on the surface of MgO after 12 MeV Si irradiation. In this way, the threshold for surface ion track formation in MgO was established between 4.2 and 5.6 keV/nm, and the upper limit for the threshold in Al_2_O_3_ and CaF_2_ has been set at 4.6 keV/nm and 3.3 keV/nm, respectively.

The results of the RBS/c analysis of sequentially irradiated samples provided evidence of defect annealing in CaF_2_ for 3 MeV O and 12 MeV O irradiations (~2.2 keV/nm). In the case of more energetic 5 MeV Si irradiation (~3.3 keV/nm), defect annealing is already counterbalanced by defect production. While Al_2_O_3_ appears not to be influenced by sequential irradiations, we found that MgO is more sensitive to high-energy ion irradiation if a small number of defects are present. This is observed for 12 MeV Si irradiation (~5.6 keV/nm), i.e., well below the threshold for ion track formation in this material (above 34 keV/nm).

The presented results clearly show that ion beams available at small accelerator facilities (like 3 MeV O and 5 MeV Si) can be used effectively in material modification and radiation hardness studies. Furthermore, damage build-up due to electronic stopping can occur in materials well below the expected thresholds. This could influence material and device behaviours in unexpected ways when operating in harsh radiation environments.

## Figures and Tables

**Table 1:** List of ion beams used in the present study. Electronic stopping *S_e_*, nuclear stopping *S_n_* and ion ranges *R* were calculated using the SRIM code [2].

Material	Density (g/cm^3^)	Ion Beam and Energy	*S_e_* (keV/nm)	*S_n_* (keV/nm)	*R* (μm)
Al_2_O_3_	3.95	600 keV I	1.24	3.21	0.13
		1.8 MeV I	1.86	2.18	0.4
		23 MeV I	8.98	0.45	4.13
		18 MeV Cu	8.98	0.01	3.8
		5 MeV Si	4.59	0.04	1.88
		12 MeV Si	5.89	0.02	3.17
		1 MeV p	0.07	0	8.99
MgO	3.58	400 keV I	1	3.22	0.1
		600 keV I	1.1	3.01	0.14
		23 MeV I	8.23	0.41	4.49
		5 MeV Si	4.21	0.04	2.02
		12 MeV Si	5.63	0.02	3.4
		1 MeV p	0.07	0	9.64
CaF_2_	3.18	1.8 MeV I	1.09	1.65	0.57
		3 MeV O	2.07	0.01	2.62
		12 MeV O	2.34	0.003	6.44
		5 MeV Si	3.27	0.03	2.78
		12 MeV Si	4.5	0.02	4.52
		1 MeV p	0.06	0	11.89

## Data Availability

Data is available on request.
